# Nontraumatic subdural hematoma in patients on hemodialysis with end-stage kidney disease: a systematic review and pooled analysis

**DOI:** 10.3389/fneur.2023.1251652

**Published:** 2023-09-15

**Authors:** Liling Yang, Zongping Li, Xiaoyu Dai, Lijun Wang, Xiaoyi Wang, Hongyuan Liu

**Affiliations:** ^1^Department of Nephrology, Mianyang Central Hospital, School of Medicine, University of Electronic Science and Technology of China, Mianyang, Sichuan, China; ^2^Department of Neurosurgery, Mianyang Central Hospital, School of Medicine, University of Electronic Science and Technology of China, Mianyang, China

**Keywords:** end-stage kidney disease, nontraumatic subdural hematoma, hemodialysis, kidney failure, kidney replacement therapy

## Abstract

**Background:**

The original treatment may aggravate when hemodialysis (HD) patients have nontraumatic subdural hematoma (NSDH). End-stage kidney disease patients are at increased risk for NSDH, but its risk factors and outcomes are not sufficiently explored at present.

**Methods:**

Electronic databases, including PubMed, EMBASE, and Web of Science were searched by using various combinations of the keywords “Hemodialysis,” “Renal Insufficiency,” “Extracorporeal Dialysis,” “Subdural Hematoma,” “Subdural Hemorrhage,” “Subdural Hematomas,” and “Subdural Hemorrhages” in accordance with the PRISMA guidelines. Sixteen papers were selected. Relevant patient data were extracted, aggregated, and analyzed.

**Results:**

A total of 74 patients were analyzed, including 37 male, 26 female, and 11 with no gender data, with a mean age of 56 years (range, 16–81 years). There were 43 patients with hypertension, 36 patients with diabetes, 16 patients who used oral anticoagulants before dialysis, and 10 patients with atrial fibrillation. The diagnosis of subdural hematoma (SDH) was made by computed tomography (CT) (*n* = 51), carotid arteriography (*n* = 7), surgical exploration (*n* = 3), and autopsy (*n* = 2). Forty cases underwent surgical treatment, including craniotomy and burr hole (or twist drill) drainage. The 1 year mortality rate of NSDH was 45.9%. The mortality rate after conservative treatment (61.8%) was higher than that after surgical intervention (32.5%). The mortality rate of NSDH in dialysis patients with atrial fibrillation and in those who used oral anticoagulants before hemodialysis (HD) was 90 and 81%, respectively.

**Conclusion:**

NSDH is rare in HD, and mortality is high if NSDH occurs in dialysis patients. Surgical intervention reduces the mortality from NSDH in patients on HD (*p* < 0.02). Patients with atrial fibrillation and those who were taking oral anticoagulants before dialysis have a higher NSDH mortality (*p* < 0.01).

## Introduction

Subdural hematoma (SDH) is a common neurosurgical condition characterized by progressive and recurrent bleeding caused by traumatic tearing of blood vessel(s) (1, 2). Patients with SDH exhibit different clinical symptoms depending on its location in the skull. SDH is classified as acute, subacute, and chronic depending on the time of development. Risk factors for developing SDH include age, head injury, anticoagulant or antiplatelet drug use, low intracranial pressure, and hemodialysis (HD) (3–5).

Chronic kidney disease is a chronic structural or functional abnormality of the kidney with various causes, and is currently a global public health problem. It eventually progresses to end-stage kidney disease (ESKD) (6), which is the final stage of chronic kidney disease and is generally irreversible.

In recent years, the incidence and mortality of ESKD have increased rapidly (7). The primary modality for treating ESKD is kidney replacement therapy (KRT), including HD, peritoneal dialysis (PD), or kidney transplantation. In ESKD patients, the accumulation of toxins leads to various complications.

Patients with ESKD have a 3–10 times higher risk of stroke than the general population (8). Because brain atrophy is common in patients with ESKD, the length of the pontine vein that is prone to tearing increases the incidence of SDH (9). Patients with long-term HD have a 10-fold higher risk of developing SDH than the general population (5, 10). This may be related to HD changes in intracranial pressure, cerebral blood flow, and subdural pressure (11). The occurrence of nontraumatic SDH (NSDH) in ESKD patients undergoing HD has rarely been reported and was mainly presented in the form of case reports or a small case series. To explore the pathophysiology and risk factors for the development of NSDH in HD, we conducted a systematic review and summary analysis of the published literature.

## Methods

The analysis and generation of inclusion criteria were based on the Preferred Reporting Items for Systematic Reviews and Meta-Analysis (PRISMA) (12).

We searched reports in English up to April 2023 in PubMed, EMBASE, and Web of Science, using Boolean operators “OR” and “AND” in combination OR alone with the following keywords: using various combinations of the keywords “Hemodialysis,” “Renal Insufficiency,” “Extracorporeal Dialysis,” “Subdural Hematoma,” “Subdural Hemorrhage,” “Subdural Hematomas,” and “Subdural Hemorrhages.”

First, relevant studies were screened by title and abstract. Second, the assessment was done after downloading the full text. Prospective studies, retrospective studies, and case reports were included. Traumatic SDH, incomplete data, non-English language reports, PD, duplicate articles, reviews, commentaries, or editorials were excluded. This process was carried out independently by three assessors (LY, ZL, and XD). Any disagreements were settled by consensus. All data were collected by two authors (LW and XW). Given that the majority were retrospective studies and individual patient data were not always available, a formal meta-analysis could not be performed.

### Statistical analysis

Statistical analysis was performed using SPSS 24.0. Univariate analysis was performed using Fisher’s exact test, and categorical variables were compared using chi-square tests. Differences were considered statistically significant at *p* values lower than 0.05.

## Results

**Figure 1:**
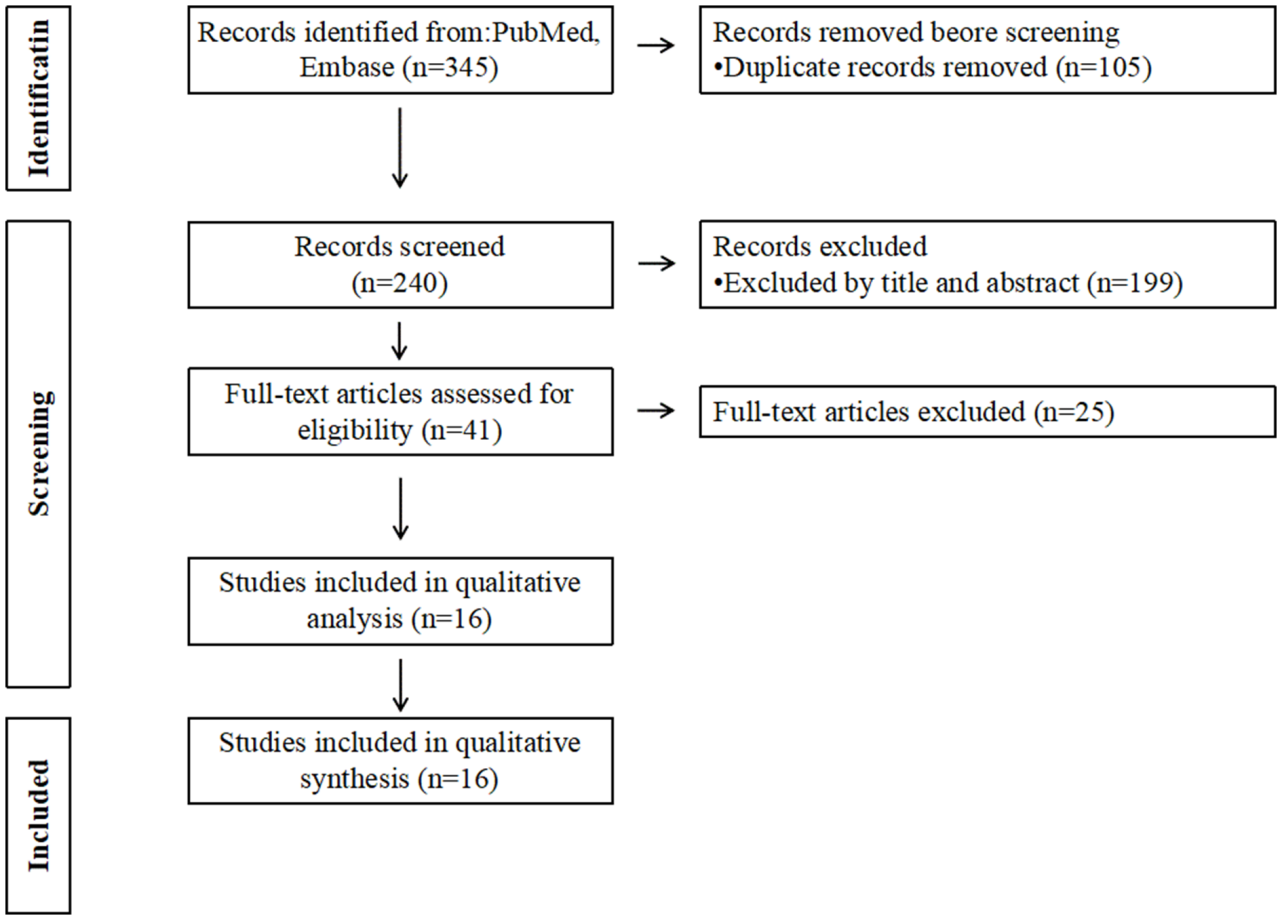
PRISMA flow diagram for the selection of literature.

By searching the keywords in the title and abstract, 105 duplicates were removed, and a total of 240 papers were obtained. Forty-one articles were obtained by filtering the titles and abstracts. The full-text versions of the 41 articles were evaluated. After excluding traumatic SDH, incomplete data, duplicate data papers, etc., a total of 16 papers were finally obtained (13–28). The PRISMA flow diagram for the selection is shown in Figure 1.

### Patient population

Sixteen papers were analyzed, with sample sizes ranging from 1 to 41 patients (Table 1). The 16 papers included 13 case reports or mini case papers (up to four patients), one article mentioning NSDH in an HD patient, and two retrospective case series (11–41 patients). A total of 74 patients were identified, namely 26 female, 37 male, and 11 with no gender data, with a mean age of 56 years (range, 16–81 years).

**Table 1:** Summary of the included studies.

Author and Year	No. of Patients	Age/Average Age (years)	Sex	Study Design	Symptoms	Symptom duration (h)	Type of SDH	Oral Anticoagulants before HD	Anticoagulants for HD	Dose of anticoagulants for HD	SDH-detection method	Treatment modalities
Prasad et al., 2022 (13)	1	27	F	Case Report	Consciousness disturbance	NA	Chronic	NA	NA	NA	CT	Levetiracetam; vitamin K; burr hole
Fayed et al., 2021 (14)	41	56.3	F (*n* = 18), M (*n* = 23)	Retrospective case series	NA	NA	Acute (*n* = 15); Chronic (*n* = 7)	Warfarin (*n* = 10)	Heparin	500 IU at the start of HD; 500 IU every hour	CT	No surgery (*n* = 19); surgery (*n* = 22)
Uchio et al., 2021 (15)	1	81	F	Case Report	Vomiting; nausea	NA	Acute	NA	NA	NA	CT	Fasting and antihypertensives
Power et al., 2010 (16)	11	71.3	NA	Retrospective case series	NA	NA	NA	NA	Heparin	500 IU at the start of HD; 500 IU every hour	NA	No surgery (*n* = 8); surgery (*n* = 3)
Mesquita et al., 2008 (17)	1	65	M	Case Report	Hemiparesis	0.2	Acute	NA	Heparin	NA	CT	Valproic acid; vancomycin; craniotomy
Sengul et al., 2005 (18)	1	26	F	Case Report	Headache; vomiting; hemiparesis	1	Acute	NA	Heparin	NA	CT	Twist drill
Zingale et al., 1999 (19)	1	77	M	Case Report	Consciousness disturbance	NA	Chronic	NA	Heparin	NA	CT	Burr hole
Kopitnik et al., 1989 (20)	1	48	M	Case Report	Consciousness disturbance	NA	Chronic	NA	Heparin	NA	CT	Burr hole
Inzelberg et al., 1989 (21)	1	28	F	Case Report	Hemiparesis	1	Chronic	Warfarin	Heparin	NA	CT	Dexamethasone; antibiotic
Sayre et al., 1987 (22)	1	29	M	Case Report	Consciousness disturbance	NA	NA	NA	Heparin	1,100 IU	CT	Craniotomy
Isiadinso et al., 1976 (23)	4	65	M	Retrospective case series	Headache	NA	Chronic	NA	Heparin	NA	Carotid arteriography	Craniotomy
Bechar et al., 1972 (24)	2	27.5	F, M	Case Report	Headache	NA	Chronic and acute	Sintrom	Heparin	NA	Carotid arteriography	Craniotomy
Talalla et al., 1970 (25)	3	29.3	F (*n* = 2), M	Retrospective case series	Headache	NA	Acute (*n* = 1); Chronic (*n* = 2)	Warfarin (*N* = 2)	Heparin	1,500 IU (*n* = 2)	Surgical exploration (*n* = 1); carotid arteriography (*n* = 1); autopsy (*n* = 1)	Burr hole and craniotomy (*n* = 1); craniotomy (*n* = 1)
Zarowny et al., 1970 (26)	1	46	M	Case Report	Headache	1.5	Acute	Anticoagulant	Heparin	NA	Surgical exploration	Burr hole
Del et al., 1970 (27)	1	28	F	Case Report	Headache	NA	NA	NA	Heparin	45–60 mg	CT	Regional heparinization
Leonard et al., 1969 (28)	3	64.3	M	Retrospective case series	Headache (*n* = 2); consciousness disturbance	NA	Acute (*n* = 2); Chronic (*n* = 1)	NA	Heparin	NA	Autopsy (*n* = 1); surgical exploration (*n* = 1); CT (*n* = 1)	Discontinuation of dialysis (*n* = 1); craniotomy (*n* = 2)

SDH, subdural hematoma; CT, computed tomography; h, hour; NA, not available.

### Kidney failure and anticoagulants

Except for one case of acute anuretic kidney failure undergoing HD, the remaining 73 patients were on chronic HD with unexplained causes of chronic kidney failure. In 14 articles, heparin anticoagulation was used during HD in 72 cases. For two females in two case reports, the authors did not mention whether heparin anticoagulation was used during HD. A total of 16 patients received oral anticoagulants before HD, while 45 cases were not anticoagulated, and the use of anticoagulants was not described in the remaining patients.

### Clinical symptoms and past complications

Two retrospective studies analyzed a total of 3,718 patients with chronic maintenance HD, including 52 patients with NSDH whose clinical symptoms at presentation were not described. The main clinical symptoms of 22 patients in 14 articles were headache (*n* = 14), consciousness disturbance (*n* = 5), hemiparesis (*n* = 3), vomiting (*n* = 2), and nausea (*n* = 1).

Of all 74 patients, 43 cases had hypertension, 36 cases had diabetes mellitus, 14 cases had ischemic heart disease, 10 cases had atrial fibrillation, three cases had chronic-disease anemia, and one case had thrombosis, and in five cases no previous diseases were mentioned.

### Auxiliary examinations

Hemoglobin level was reported in 59 patients in eight articles, with a mean of 106.4 g/L. Prothrombin time was reported in 55 patients in five articles, with an average of 11.74 s. The mean platelet in 55 patients in five articles was 181.27 × 10^9^/L.

The diagnosis of SDH was made by computed tomography (CT) in 51 cases, carotid arteriography in seven cases, surgical exploration in three cases, and autopsy in two cases. Twenty-three cases were acute SDH, 16 cases were chronic SDH, and the nature of SDH was unclear in 35 cases.

### Treatment and outcome

Thirty-four cases underwent conservative treatment, such as antihypertensive therapy, anti-epilepsy, and change of anticoagulant for HD, including one case where conservative treatment was followed by burr hole and one case where it was followed by craniotomy. Forty cases underwent surgical treatment, including craniotomy and burr hole (or twist drill) drainage. The 30 days mortality rate of conservative treatment was 61.8%, while the 30 days mortality rate of surgical treatment was 30%. At 1 year, 40 patients survived.

### Risk factors for death

Table 2 presents the data on survival and death of the patients with NSDH who underwent HD within 1 year. In the survivor group, 27 (67.5%) of 40 patients underwent surgical intervention, while in the death group, 13 (38.2%) of 34 cases underwent surgical intervention. Aggressive surgical intervention for NSDH on HD was able to reduce the risk of death (*p* < 0.02). Patients with atrial fibrillation or those who were taking oral anticoagulants before HD had increased mortality from SDH (*p* < 0.01). Age, sex, hypertension, diabetes, and type of SDH did not change the outcome of NSDH on HD.

**Table 2:** Risk factors affecting the survival rate of SDH on HD.

	Survival (*n* = 40)	Death (*n* = 34)	*p*
Age (years)	52.2 (*n* = 35)	62.4 (*n* = 24)	NS
Male Sex	20/36 (55.6%)	17/27 (62.9%)	NS
Acute subdural hematomas	16/28 (57.1%)	7/14 (50%)	NS
Surgical treatment	27/40 (67.5%)	13/34 (38.2%)	0.02
Diabetes mellitus	19/33 (57.6%)	12/22 (54.5%)	NS
Hypertension	21/34 (64.7%)	15/21 (71.4%)	NS
Atrial fibrillation	1/33 (3%)	9/22 (40.9%)	0.001
Oral anticoagulant medication before HD	3/36 (8.3%)	13/25 (52%)	0.001

SDH, subdural hematoma; HD, hemodialysis.

## Discussion

Stroke and ESKD are two major risk factors for human health. There is a high risk of stroke in patients with ESKD undergoing dialysis and a high mortality rate in the event of stroke. SDH usually occurs in injured elderly patients, with high morbidity and mortality rate. Fall, age, antiplatelet drugs, and low intracranial pressure are risk factors for the development of SDH (3, 4). Among them, fall and age are common clinical risk factors. In the absence of trauma, SDH is thought to occur spontaneously and may be associated with systemic hypertension, cerebral atrophy, coagulation dysfunction, and use of anticoagulant drugs. Some studies have shown that dialysis modality may affect the incidence of SDH in ESKD (29–32). HD alters hemodynamics, and the use of anticoagulants such as heparin puts HD patients at a higher risk of SDH than PD patients (33). Some studies have shown that HD patients have a higher rate of SDH (5, 10, 34). In two of the studies we collected, 52 out of 3,718 patients developed SDH, with an incidence of 1.4% and an annual incidence of 189 per 100,000/year. By contrast, previous studies have reported an incidence of SDH between 8.2/100,000 and 48/100,000/year (29). The high incidence of NSDH in HD may be related to heparin and other anticoagulants, antiplatelet drugs, atrial fibrillation, and cardiovascular diseases (14). It has also been shown that the increased risk of HD bleeding is due to the uremic state itself and impaired platelet function (14, 33). Although the incidence of NSDH is higher in HD patients, there are few literature reports, and the previous reports were mainly case reports. With insufficient data on the risk factors and outcomes, we conducted a systematic review of previous HD patients with NSDH.

The onset of NSDH is relatively insidious, and the common clinical symptoms are headache, consciousness disturbance, and hemiparesis. In our report, the symptoms included headache (*n* = 14), consciousness disturbance (*n* = 5), hemiparesis (*n* = 3), vomiting (*n* = 2), and nausea (*n* = 1). The diagnosis of NSDH was mainly made by imaging, among which 51 cases were found by CT examination and seven cases were found by carotid arteriography. However, before the development and popularization of CT, NSDH was usually detected at autopsy or surgical exploration. In our retrospective analysis, two cases were found by autopsy and three cases by surgical exploration. The treatment of NSDH is similar to that of traumatic SDH. Treatment of SDH usually depends on the patient’s symptoms, neurological examination, thickness of blood in the SDH, midline shift, and other factors (35–37). Surgical intervention for SDH is the preferred treatment for patients with symptoms and/or midline displacement >1 cm, or with supratentorial hematoma greater than 30 mL. For subacute or chronic SDH, only external drainage is usually required. However, the burr hole or twist drill should be wide enough to allow continuous free drainage to prevent recurrence of the hematoma. There were no reports of recurrence after burr hole or twist drill in our study. For acute SDH, craniotomy is usually required to determine the site of bleeding. The timing of surgery is critical for acute SDH in patients receiving anticoagulant therapy. Some studies have shown that mortality from early surgery is nearly twice as high as that from late surgery due to coagulopathy and inadequate preparation for surgical intervention (38). In our study, there were 19 cases of acute SDH craniotomy and five cases of death.

In past retrospective studies, it has been reported that the 30 days mortality rate for SDH patients with ESKD requiring dialysis is 19% (34). Two SDH surveys of ESKD patients in Taiwan and the United States have reported mortality rates of 35–39% (5, 39). The 30 days mortality rate for conservative treatment was 61.8%, and the mortality rate for surgical treatment was 30%. The one-year mortality rate was 45.9%. This is broadly consistent with previous reports. We further analyzed the risk factors for death after the development of SDH in HD patients. Surgical intervention, atrial fibrillation, and oral anticoagulants before HD can change the outcome of SDH. Surgical intervention after the occurrence of NSDH in HD can reduce patient mortality (*p* < 0.02). Consistent with other studies (39, 40), we confirmed that atrial fibrillation is associated with an increased risk of death from NSDH in HD (*p* < 0.01). At the same time, our study showed that oral anticoagulants prior to HD increased the risk of death from NSDH (*p* < 0.01). It is possible that the use of oral anticoagulants in the management of atrial fibrillation may exacerbate uremic bleeding (41, 42). However, variables such as age, hypertension, and diabetes do not change the outcome of NSDH occurring in HD.

There are limitations to our study. First, the vast majority of the studies reviewed were retrospective and observational, and such studies are prone to selection and publication bias. Therefore, the strength of our data and the validity of our conclusions are limited. Second, not every data variable could be extracted due to the design of the study and the heterogeneity of the published data. Nevertheless, we were the first to conduct a comprehensive and systematic review of the literature on the occurrence of NSDH in HD patients and to assess the incidence of NSDH in HD and the risk factors for death.

## Conclusion

NSDH is rare in HD patients, but has the potential to be a serious complication, with a possible mortality rate of 39–45%. If NSDH occurs on HD, conservative treatment is associated with a twofold increased risk of death compared with surgical intervention. Patients with atrial fibrillation or those who were taking oral anticoagulants before dialysis have a greater risk of death when NSDH occurs during dialysis. Given the significance of our findings, prospective studies may be needed to help accurately determine the incidence, risk factors, and outcomes of this complication to develop effective prevention and treatment strategies for this population.

## Data availability statement

The original contributions presented in the study are included in the article/Supplementary material, further inquiries can be directed to the corresponding author.

## Author contributions

LY and HL: conceptualization. XW and HL: methodology and supervision. ZL, XD, and LW: data curation. LY and ZL: writing – original draft preparation. HL: writing – review and editing and project administration. All authors contributed to the article and approved the submitted version.

## Funding

This work was supported by the Foundation Program of the Sichuan Provincial Health Commission (no. 21PJ181).

## Conflict of interest

The authors declare that the research was conducted in the absence of any commercial or financial relationships that could be construed as a potential conflict of interest.

## Publisher’s note

All claims expressed in this article are solely those of the authors and do not necessarily represent those of their affiliated organizations, or those of the publisher, the editors and the reviewers. Any product that may be evaluated in this article, or claim that may be made by its manufacturer, is not guaranteed or endorsed by the publisher.
