# Supplementary material for: Nontraumatic subdural hematoma in patients on hemodialysis with end-stage kidney disease: a systematic review and pooled analysis
Source: Front Neurol. 2023 Sep 15;14:1251652. doi: 10.3389/fneur.2023.1251652 (PMC10542405; doi:10.3389/fneur.2023.1251652)
Supplement: Supplementary file 1 [file Data_Sheet_1.docx]

Table 1. Summary of the included studies.

| Author and Year | No. of Patients | Age/  Average Age  (years) | Sex | Study Design | Symptoms | Symptom duration (h) | Type of SDH | Oral Anticoagulants before HD | Anticoagulants  for HD | Dose of anticoagulants  for HD | SDH-Detection  Method | Treatment modalities |
| --- | --- | --- | --- | --- | --- | --- | --- | --- | --- | --- | --- | --- |
| Prasad et al., 2022^13^ | 1 | 27 | F | Case Report | Consciousness disturbance | NA | Chronic | NA | NA | NA | CT | Levetiracetam;  vitamin K;  burr hole |
| Fayed et al., 2021^14^ | 41 | 56.3 | F (n=18),  M (n=23) | Retrospective  Case Series | NA | NA | Acute (n=15);  Chronic (n=7) | Warfarin (n=10) | Heparin | 500 IU at the start of HD;  500 IU every hour | CT | No surgery (n=19);  surgery (n=22) |
| Uchio et al., 2021^15^ | 1 | 81 | F | Case Report | Vomiting;  nausea | NA | Acute | NA | NA | NA | CT | Fasting and antihypertensives |
| Power et al., 2010^16^ | 11 | 71.3 | NA | Retrospective  Case Series | NA | NA | NA | NA | Heparin | 500 IU at the start of HD;  500 IU every hour | NA | No surgery (n=8);  surgery (n=3) |
| Mesquita et al., 2008^17^ | 1 | 65 | M | Case Report | Hemiparesis | 0.2 | Acute | NA | Heparin | NA | CT | Valproic acid;  vancomycin;  craniotomy |
| Sengul et al., 2005^18^ | 1 | 26 | F | Case Report | Headache;  vomiting;  hemiparesis | 1 | Acute | NA | Heparin | NA | CT | Twist drill |
| Zingale et al., 1999^19^ | 1 | 77 | M | Case Report | Consciousness disturbance | NA | chronic | NA | Heparin | NA | CT | Burr hole |
| Kopitnik et al., 1989^20^ | 1 | 48 | M | Case Report | Consciousness disturbance | NA | Chronic | NA | Heparin | NA | CT | Burr hole |
| Inzelberg et al., 1989^21^ | 1 | 28 | F | Case Report | Hemiparesis | 1 | Chronic | Warfarin | Heparin | NA | CT | Dexamethasone; antibiotic |
| Sayre et al., 1987^22^ | 1 | 29 | M | Case Report | Consciousness disturbance | NA | NA | NA | Heparin | 1100 IU | CT | Craniotomy |
| Isiadinso et al., 1976^23^ | 4 | 65 | M | Retrospective  Case Series | Headache | NA | Chronic | NA | Heparin | NA | Carotid arteriography | Craniotomy |
| Bechar et al., 1972^24^ | 2 | 27.5 | F,  M | Case Report | Headache | NA | Chronic and acute | Sintrom | Heparin | NA | Carotid arteriography | Craniotomy |
| Talalla et al., 1970^25^ | 3 | 29.3 | F (n=2), M | Retrospective  Case Series | Headache | NA | Acute (n=1);  Chronic (n=2) | Warfarin (N=2) | Heparin | 1500 IU (n=2) | surgical exploration (n=1);  carotid arteriography (n=1);  autopsy (n=1) | Burr hole and craniotomy (n=1); craniotomy (n=1) |
| Zarowny et al., 1970^26^ | 1 | 46 | M | Case Report | Headache | 1.5 | Acute | Anticoagulant | Heparin | NA | Surgical exploration | Burr hole |
| Del et al., 1970^27^ | 1 | 28 | F | Case Report | Headache | NA | NA | NA | Heparin | 45–60 mg | CT | Regional heparinization |
| Leonard et al., 1969^28^ | 3 | 64.3 | M | Retrospective  Case Series | Headache (n=2);  consciousness disturbance | NA | Acute (n=2);  Chronic (n=1) | NA | Heparin | NA | Autopsy (n=1);  surgical exploration (n=1);  CT (n=1) | Discontinuation of dialysis (n=1); craniotomy (n=2) |

SDH: Subdural hematoma; CT: computed tomography; h: hour; NA: not available.

Table 2. Risk factors affecting the survival rate of SDH on HD

|  | Survival (n=40) | Death (n=34) | p |
| --- | --- | --- | --- |
| Age (years) | 52.2 (n=35) | 62.4 (n=24) | NS |
| Male Sex | 20/36 (55.6%) | 17/27 (62.9%) | NS |
| Acute subdural hematomas | 16/28 (57.1%) | 7/14 (50%) | NS |
| Surgical treatment | 27/40 (67.5%) | 13/34 (38.2%) | 0.02 |
| Diabetes mellitus | 19/33 (57.6%) | 12/22 (54.5%) | NS |
| Hypertension | 21/34 (64.7%) | 15/21 (71.4%) | NS |
| Atrial fibrillation | 1/33 (3%) | 9/22 (40.9%) | 0.001 |
| Oral anticoagulant medication before HD | 3/36 (8.3%) | 13/25 (52%) | 0.001 |

SDH: Subdural hematoma, HD: hemodialysis.
